# Nox2 Upregulation and p38α MAPK Activation in Right Ventricular Hypertrophy of Rats Exposed to Long-Term Chronic Intermittent Hypobaric Hypoxia

**DOI:** 10.3390/ijms21228576

**Published:** 2020-11-13

**Authors:** Eduardo Pena, Patricia Siques, Julio Brito, Silvia M. Arribas, Rainer Böger, Juliane Hannemann, Fabiola León-Velarde, M. Carmen González, M. Rosario López, Ángel Luis López de Pablo

**Affiliations:** 1 Institute of Health Studies, Universidad Arturo Prat, Avenida Arturo Prat 2120, Iquique 1110939, Chile; psiques@tie.cl (P.S.); jbritor@tie.cl (J.B.); 2 Institute DECIPHER, German-Chilean Institute for Research on Pulmonary Hypoxia and Its Health Sequelae, Hamburg (Germany) and Iquique (Chile), Avenida Arturo Prat 2120, Iquique 1110939, Chile; boeger@uke.de (R.B.); j.hannemann@uke.de (J.H.); 3 Department of Physiology, University Autónoma of Madrid, Calle Arzobispo Morcillo 4, 28029 Madrid, Spain; silvia.arribas@uam.es (S.M.A.); m.c.gonzalez@uam.es (M.C.G.); angel.lopezdepablo@uam.es (Á.L.L.d.P.); 4 Institute of Clinical Pharmacology and Toxicology, University Medical Center Hamburg-Eppendorf, Martinistraße 52, 20246 Hamburg, Germany; 5 Department of Biological and Physiological Science, Facultad de Ciencias y Filosofía/IIA, Cayetano Heredia University, Avenida Honorio Delgado 430, Lima 15102, Peru; fleon-velarde@concytec.gob.pe; 6 Department of Preventive Medicine and Public Health, University Autónoma of Madrid, Calle Arzobispo Morcillo 4, 28029 Madrid, Spain; mrosario.lopez@uam.es

**Keywords:** oxidative stress, high altitude, cardiac hypertrophy, kinases and NADPH oxidase

## Abstract

One of the consequences of high altitude (hypobaric hypoxia) exposure is the development of right ventricular hypertrophy (RVH). One particular type of exposure is long-term chronic intermittent hypobaric hypoxia (CIH); the molecular alterations in RVH in this particular condition are less known. Studies show an important role of nicotinamide adenine dinucleotide phosphate (NADPH) oxidase complex-induced oxidative stress and protein kinase activation in different models of cardiac hypertrophy. The aim was to determine the oxidative level, NADPH oxidase expression and MAPK activation in rats with RVH induced by CIH. Male Wistar rats were randomly subjected to CIH (2 days hypoxia/2 days normoxia; n = 10) and normoxia (NX; n = 10) for 30 days. Hypoxia was simulated with a hypobaric chamber. Measurements in the RV included the following: hypertrophy, Nox2, Nox4, p22phox, LOX-1 and HIF-1α expression, lipid peroxidation and H_2_O_2_ concentration, and p38α and Akt activation. All CIH rats developed RVH and showed an upregulation of LOX-1, Nox2 and p22phox and an increase in lipid peroxidation, HIF-1α stabilization and p38α activation. Rats with long-term CIH-induced RVH clearly showed Nox2, p22phox and LOX-1 upregulation and increased lipid peroxidation, HIF-1α stabilization and p38α activation. Therefore, these molecules may be considered new targets in CIH-induced RVH.

## 1. Introduction

Exposure of individuals to high altitude usually results in decreased oxygen bioavailability in blood and tissues (hypobaric hypoxia) [1].

Altitude hypobaric hypoxia is classified according to the time of exposure, and there are three well-determined conditions: chronic hypobaric exposure, which represents people who live permanently at high altitude (Andes and Himalayas) [2] and acute exposure, which represents people who are exposed to high altitude for hours or few days (tourists and climbers). However, of particular interest is the growing number of individuals who are subjected to periods of work (days) at high altitude and rest (days) at sea level for years. This condition is completely different from other types of intermittent hypobaric hypoxia exposure (hours or seconds), such as in obstructive sleep apnea, training, and exercise, among other conditions [3]. Initially developed by studying mining activities in the north of Chile involving a great number of workers who were intermittently exposed to high altitude (3800 to 4600 m) for a long period of time, this condition is currently known throughout the world as “long-term chronic intermittent hypobaric hypoxia” exposure (CIH) [3,4].

Several studies have clearly established that hypobaric hypoxia exposure induces pulmonary hypertension [5,6,7] due to hypoxic pulmonary artery vasoconstriction [8,9], causing pulmonary artery remodeling and the subsequent development of pressure overload (PO)-induced right ventricular hypertrophy (RVH) [10,11,12]. In addition, this phenomenon has also been described in long-term CIH [13,14,15]. However, the molecular mechanisms underlying the development of RVH under this particular condition have yet to be determined.

Previous studies in animals [16] and humans [17] show that hypobaric hypoxia exposure induces oxidative stress in the heart. Moreover, oxidative stress has emerged as an important transducer of PO-induced cardiac hypertrophy [18,19,20]. In fact, several studies show that both hypobaric hypoxia and oxidative stress can activate kinase proteins, such as Akt and p38α MAPK, and stabilize hypoxia-inducible transcription factor-1α (HIF-1α) [21,22], which are related to cardiac hypertrophy and ventricular remodeling [23,24,25,26].

One important contributor to oxidative stress in the heart is the nicotinamide adenine dinucleotide phosphate (NADPH) oxidase complex [20,27]. It is composed of seven catalytic isoforms termed Nox1 to Nox5 and Duox1/2 [28]. These isoforms of Nox are distinct according to their distribution in vivo, expression in cell types, locations in subcellular compartments, products, and functions in physiology and pathology [29]. For example, Nox2 and Nox4 isoforms are primarily expressed in the heart, with their p22phox subunit [18,27,30]. When these complexes (Nox) are activated, oxidative stress is produced through the exacerbated production of reactive oxygen species (ROS), which could result in damage to the cellular structure, such as lipid peroxidation [31]. However, it is important to highlight that Nox4 is constitutively active and is the only isoform of the Nox family involved in production of hydrogen peroxide (H_2_O_2_) as the main product [29]. The rest of Nox isoforms (Nox1, Nox2, Nox3, Nox5, Doux1 and Doux2) produce superoxide (O_2_^−^) as the principal product [32], while Duox1/2 also produce H_2_O_2_ [33].

Despite this difference regarding oxidative products, it has been reported that Nox4 and Nox2 upregulation is related to cardiac hypertrophy [29]. This is supported by recent studies specifically involving RVH induced by other models of hypoxia (normobaric hypoxia) showing an upregulation of Nox2 and Nox4 through the activation of lectin-like oxidized low-density lipoprotein receptor-1 (LOX-1) [20]. These data highlight that LOX-1 can be activated not only by hypoxia but also by oxidized lipoprotein and shear stress [34,35]. Therefore, we hypothesized that Noxs (Nox2/4), p38αMAPK and Akt kinase, HIF-1α and LOX-1 would be overexpressed in CIH-induced RVH in rats, which we assessed in rats exposed to long-term CIH.

## 2. Results

### 2.1. Hematocrit and Ventricular Hypertrophy

At the end of the exposure period, the CIH rats exhibited higher Hct levels (64.3 ± 1.9) than the NX group (44.1 ± 2.3; *p* < 0.05) as expected.

**Figure 1:**
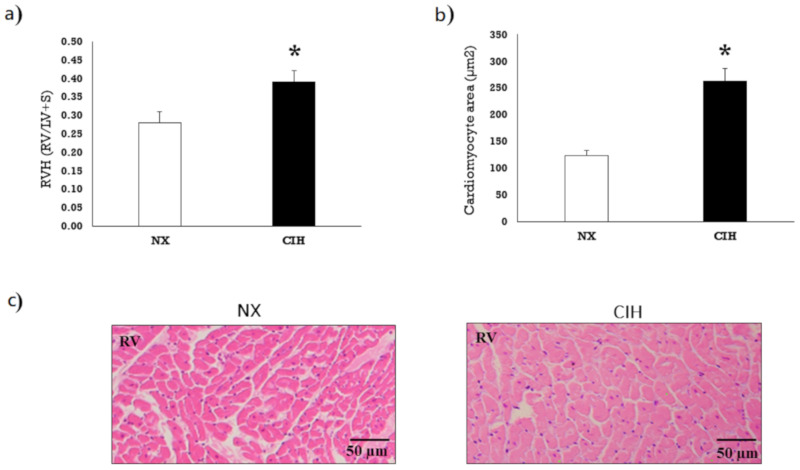
(**a**) Degree of right ventricle hypertrophy (RVH) by Fulton’s index (right ventricle (RV)/left ventricle plus septum (LV+S)) after exposure to normoxia (NX; *n* = 10) and chronic intermittent hypobaric hypoxia (CIH; *n* = 10); (**b**) Cardiomyocyte area in the RV (µm^2^); (**c**) Representative image of hematoxylin-eosin-stained RV tissue. The values are the mean ($\bar{x}$) ± standard error (SE). * *p* < 0.05: CIH group vs. NX group.

The rats under CIH conditions showed significantly larger Fulton’s index values than the rats under NX conditions (*p* < 0.05), which indicated the presence of RVH (Figure 1a). H&E staining revealed significantly enlarged right ventricular cardiomyocytes in the CIH group, supporting Fulton’s index findings (Figure 1b,c).

### 2.2. LOX-1, Nox2, Nox4 and p22phox Expression

**Figure 2:**
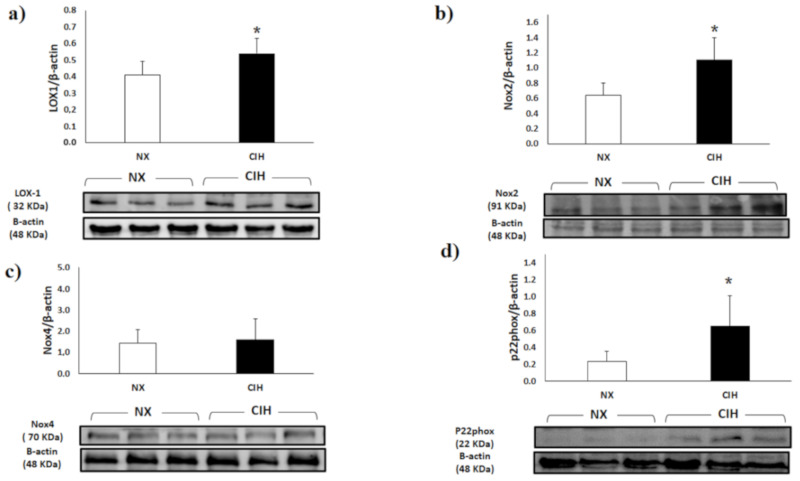
Expression of (**a**) Lectin-like oxidized low-density lipoprotein receptor-1 (LOX-1); (**b**) Nicotinamide adenine dinucleotide phosphate oxidase-2 (Nox2); (**c**) Nicotinamide adenine dinucleotide phosphate oxidase-4 (Nox4); (**d**) p22phox subunit (p22phox) expression in the right ventricle (RV) in the normoxia (NX); *n =* 10 and chronic intermittent hypobaric hypoxia (CIH); n = 10, groups, normalized by β-actin expression. Representative bands are shown. The values are the mean ($\bar{x}$) ± standard error (SE). * *p* < 0.05: CIH group vs. NX group.

LOX-1 expression in the RV in the CIH group was moderately elevated compared to that in the NX group (*p* < 0.05) (Figure 2a). Nox2 expression in the RV was higher in the CIH group than in the NX group (*p* < 0.05) (Figure 2b). The protein expression of Nox4 in RV showed no differences among the groups (Figure 2c). Regarding the regulatory proteins, p22phox was significantly elevated in the CIH group (*p* < 0.05) compared to the NX group (Figure 2d).

### 2.3. Lipid Peroxidation (MDA) Level and H_2_O_2_ Concentration

**Figure 3:**
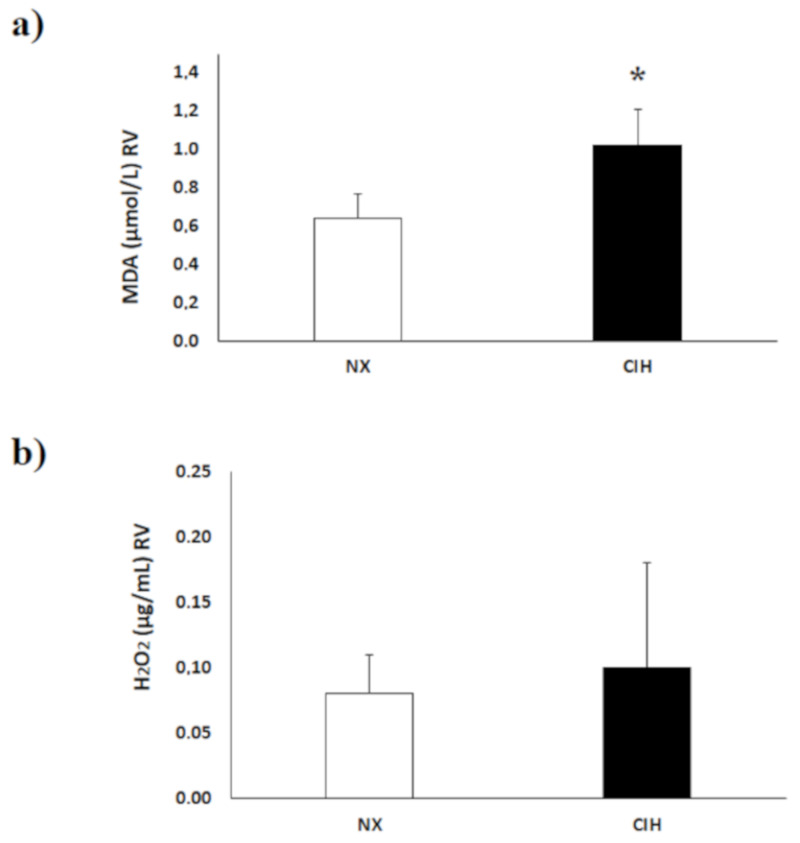
(**a**) Malondialdehyde (MDA) concentrations in the right ventricle tissue (RV); (**b**) hydrogen peroxide (H_2_O_2_) concentration in the RV under normoxia (NX); *n* = 10 and chronic intermittent hypobaric hypoxia (CIH); *n* = 10. The values are the mean ($\bar{x}$) ± standard error (SE). * *p* < 0.05: CIH vs. NX group.

The concentration of lipid peroxides, expressed as the level of malondialdehyde (MDA), was higher in the RV tissue of the CIH group than in that of the NX group (*p* < 0.05) (Figure 3a). The hydrogen peroxide (H_2_O_2_) concentration in the RV tissue showed no differences between the groups; *p* = NS (Figure 3b).

### 2.4. p38α MAPK, Akt and HIF-1α

**Figure 4:**
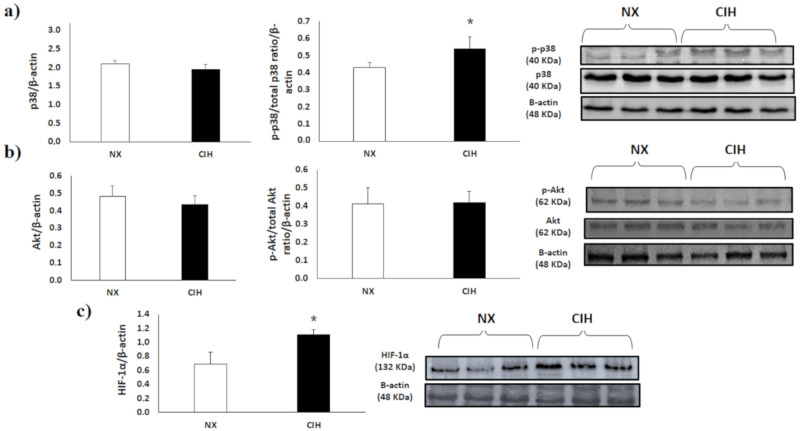
(**a**) Total p38α expression and p38α activity, assessed by the p-p38α/p38α ratio; (**b**) Total Akt expression and Akt activity assessed by the p-Akt/Akt ratio; (**c**) HIF-1α expression in the right ventricle (RV), normoxia (NX; *n* = 10), chronic intermittent hypobaric hypoxia (CIH); *n* = 10. Protein levels were normalized to β-actin expression. Representative bands are shown. The values are the mean ($\bar{x}$) ± standard error (SE). * *p* < 0.05: CIH vs. NX group.

p38α MAPK activity in the RV of the CIH rat group was increased compared with that in the RV of the NX group (Figure 4a); The percentage of p38 activation in the CIH group was 54.1%, and that in the NX group was 43.4% (*p* < 0.05). Akt activation showed no differences among groups (Figure 4b); The percentage of Akt activation in CIH was 41.9%, and that in the NX group was 40.8%. On the other hand, HIF-1α protein expression was moderately increased in the CIH group compared with the NX group (*p* < 0.05) (Figure 4c).

## 3. Discussion

We explored some molecular pathways involved in RVH under CIH conditions. The results of this study show that exposure to CIH induces RVH concomitantly with increased lipid peroxidation, LOX-1, Nox2, p22phox and HIF-1α expression, and p38α MAPK activity.

Our first finding was the increase in Hct and development of RVH, which have been found mainly in chronic and lately in intermittent hypoxia [36,37,38,39,40]. In addition, an elevated Hct level has been described as an important factor in the development of RVH by an increase in viscosity [41]. In CIH, these findings of an increased Hct and RVH have also been described, but there is a paucity of data in the literature [40,42,43].

The role of oxidative stress in cardiac hypertrophy and some specific molecular mechanisms implicated have a growing body of evidence [11,20]. This current study showed an increased MDA concentration in CIH-induced RVH, which is consistent with the findings of other studies both in animals and humans in other types of hypoxia [16,17,44,45,46], highlighting increased lipid peroxidation. In addition, the levels of MDA or lipid peroxidation measures have been described as indirect but effective indexes of ROS generation [47]. Moreover, MDA interacts with kinases involved in cardiac hypertrophy, as will be discussed later.

As mentioned before, a study established an increase in Nox2 and Nox4 expression in cardiac hypertrophy under chronic normobaric hypoxia [20]. However, our results in CIH-induced RVH show an increase in Nox2 isoform expression and its essential component, p22phox, but there were no changes in Nox4 expression, which is not only different but also a novelty in the knowledge on RVH. The unchanged Nox4 expression level was concordant with the H_2_O_2_ level in RVH since Nox4 is the only isoform of the Nox family for the constitutive production of H_2_O_2_ [29,48]. However, the finding in this current study that hypertrophic right cardiac tissue did not show changes in Nox4 expression could be explained by studies that demonstrated that Nox4 is capable of inhibiting cardiac hypertrophy by stimulating autophagy [26,49]. Moreover, it has been described that Nox4 is more prevalent in smooth muscle cells of the vascular system than in cardiac tissue [18,50,51,52].

Regarding the increased levels of MDA (lipid peroxidation) and not H_2_O_2_, this could be due to the Harber-Weiss reaction, which is activated in different cell lines under oxidative stress conditions, producing an increase in hydroxyl radicals (OH) [53,54]. In addition, this OH is the principal molecule that triggers the initiation of lipid peroxidation production through nonsaturated lipids [55], generating an increase in MDA.

On the other hand, a study showed that the deletion of Nox2 decreased oxidative stress and HIF-1α expression in cardiomyocyte cultures under normobaric hypoxia [56]. Although the latter study evaluated another type of hypoxia, it might support our findings regarding the upregulation of Nox2 and HIF-1α in RVH under CIH conditions.

Regarding the possible participation of LOX-1 as a cardiac hypertrophy inducer in CIH-induced RVH, it has been suggested that LOX-1 expression can be upregulated by multiple factors, such as hypoxia, oxidized low-density lipoprotein and shear stress [57]. Moreover, a recent study showed that in chronic normobaric hypoxia-induced RVH the levels of NADPH oxidase were strongly increased through LOX-1 [20], which could support the increased expression of LOX-1 and Nox2 found in RVH under this particular type of hypoxia.

p38α MAPK and Akt have been identified as redox-sensitive kinases and as main mediators of cardiac hypertrophy [58,59,60]. However, our data do not show Akt activation, which could suggest that this protein is not related to RVH under this particular exposure. On the other hand, the hypertrophied RV under study highlights an increase in p38α MAPK activity. This result is consistent with studies showing an increase in p38α activity through an increase in lipid peroxidation in other hypoxia models [61,62]. It is important to highlight that p38 presents four isoforms, α, β, γ (also known as Erk6 or SAPK3), and δ (also known as SAPK4), and their expression depends on the tissue and the cardiac process, where p38α has been related to cardiac ventricular hypertrophy [63]. In addition, a study in mice under other types of hypoxia (chronic normobaric hypoxia, 10% O_2_) showed that animals with PO-induced RVH exhibited increased p38α expression, which was related to several effects, such as increased collagen and smooth muscle actin α (α-SMA) content leading to cardiac fibrosis [64].

In our current study, HIF-1α protein was stabilized in the RVH induced by CIH. We hypothesized that HIF-1α stabilization found in this experimental model could be related to p38α activation since studies have shown that HIF-1α stabilization in RVH under hypoxic conditions is due to p38α activation [65,66]. In addition, the activation of p38 MAPK could be mediated by lipid peroxidation-produced MDA [61]. However, to corroborate this hypothesis, more studies are needed.

On the other hand, studies involving other hypoxic conditions (chronic hypobaric hypoxia) have shown that the activation of PKCε (Ca^2+^-independent PKC isoform) represents an adaptive and protective role, abolishing mitochondrial impairment in the heart tissue [67]. However, a study in rats with RVH induced by the same hypoxic condition (chronic hypobaric hypoxia) showed an increase in the activation of PKCα [68], where PKCα produced an increase in gelactin-3 expression, which is related to cardiac fibrosis and heart failure [69]. Therefore, the assessment of the mechanistic activity and expression of PKC isoforms in RVH under this particular condition (CIH) is very interesting for future studies. In addition, regarding this type of condition, an interesting study by Brown et al. [70] showed that mice with RVH induced by chronic hypobaric hypoxia showed a relation of MAP kinase kinase kinase-2 (MEKK-2) and the ERK5 pathway, leading to an increase in inflammatory molecules that triggered the cardiac hypertrophy process.

Moreover, it is important to highlight that a recent study in rats exposed to a chronic hypobaric hypoxia indicate that the RV differed from the left ventricle both in immune cells and expression of certain genes; therefore, this suggests that the two ventricles differ in aspects of pathophysiology and in potential therapeutic targets for RV dysfunction [71]. While these studies are not fully comparable to the hypoxic conditions in the current study, they provide new avenues to surmise hypothetical similar changes under CIH and future targets of study.

Regarding metabolic factors, two interesting studies by Muthuramu et al. [72,73] in another hypertrophy model, transverse aortic constriction (TAC), showed through cholesterol and homocysteine lowering gene therapy the pivotal role of cholesterol and homocysteine levels in the development of metabolic cardiac hypertrophy, fibrosis and heart function related to oxidative stress and protein kinase activation. Therefore, it is important to consider these pathways mentioned above in future studies since they could be present in the development of RVH induced under this particular condition of hypobaric hypoxia.

Our study attempted to present a preliminary overview of some key proteins that could be involved in long-term CIH-induced RVH compared to NX. We evidenced the presence of redox activity and some alterations in the main proteins known to be involved in cardiac hypertrophy. The information provided is rather novel because, to our knowledge, there is no information about the oxidative level and p38 activation in this particular kind of exposure to hypoxia in RVH.

**Figure 5:**
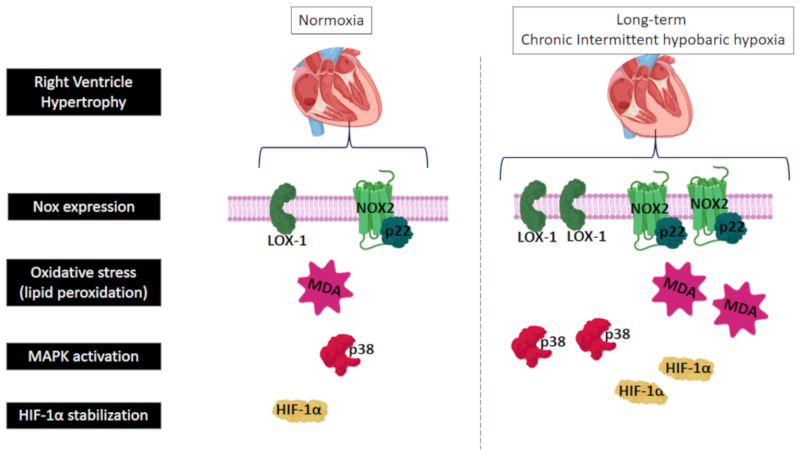
Schematic diagram of the main results and the proposed signaling pathway implicated in the development of right ventricle hypertrophy (RVH) induced by long-term intermittent hypobaric hypoxia; LOX-1: lectin-like oxidized low-density lipoprotein receptor-1; Nox2: nicotinamide adenine dinucleotide phosphate oxidase-2; p22: p22phox subunit of NADPH-oxidases; MDA: malondialdehyde; HIF-1α: hypoxia-inducible factor-1α, and p38α: mitogen-activated protein kinase p38α.

Finally, a schematic diagram of the proposed signaling pathway that might be involved in the development of RVH induced by long-term intermittent hypobaric hypoxia exposure, is shown in Figure 5.

In conclusion, long-term CIH-induced RVH seems to be mediated by oxidative stress and redox signaling due to elevated lipid peroxidation, LOX-1, Nox2 and p22phox expression, p38α MAPK activation, and HIF-1α stabilization. These findings expand the general knowledge about protein alterations during CIH-induced RVH and open new avenues for the study of RVH in this particular hypoxic condition.

## 4. Materials and Methods

### 4.1. Study Groups

Twenty male Wistar rats (3 months old) were obtained from the animal facility of the Institute of Health Studies of Arturo Prat University, Iquique, Chile. The animal protocols and all other procedures were performed in accordance with the ethical standards for the handling of experimental animals (Chilean Law 20.380, Art 7, 3 Oct 2009) and were approved by the Research Ethics Committee of Arturo Prat University, Iquique, Chile (3 Jun 2015).

Rats were placed in individual cages at a temperature of 22 ± 2 °C with a circadian rhythm of 12 h of light and 12 h of darkness. Feeding consisted of 15 g/day of food that contained 22.0% crude protein, 5.0% crude fiber, 9.0% ash and 12% moisture (5POO ^®^, LabDiet, Prolab RMH3000), and water was given ad libitum. Movement inside the cage was not restricted, but no exercise was performed.

The rats were randomly distributed into 2 experimental groups: a normobaric normoxia (NX) group, which served as the sea level control group (n = 10), and a CIH group with 2 days of exposure to hypobaric hypoxia alternating with 2 days of exposure to NX (n = 10).

### 4.2. Animal Model

The exposure time for each group was 30 days, and hypobaric hypoxia was established in a hypobaric chamber at 428 Torr, which is equivalent to the pressure at an altitude of 4,600 m above sea level (the time to reach the final pressure was 60 min). In the chamber, the internal air flow rate was 3.14 L/min, and the humidity was between 21 and 30%. The NX group rats were kept in the same chamber room under the same environmental conditions except for hypobaric hypoxia. This protocol was previously validated, and it is a model that, despite the limitations of animal models, more closely mimics the conditions of exposure to long-term CIH in humans [40,74]. All experimental procedures were performed at the end of the 30-day exposure time. On day 30, the rats were anesthetized with ketamine (0.6 mg/kg BW) to obtain blood samples. Thereafter, the rats were euthanized through fatal thoracotomy, and the hearts were removed.

### 4.3. Biomedical Variables

Body weight (BW; g) and hematocrit percentage (Hct; %) were measured at the beginning and end of the exposure period. BW was measured with an electronic balance (Acculab V-1200 ^®^, Lake Country, Illinois, USA). Blood samples were obtained by cardiac puncture in rats under anesthesia (0.3 mg of ketamine/kg BW) in heparinized vials. To determine the Hct, 1 mL of blood was transferred to a glass capillary tube and centrifuged at 5000 rpm for 5 min at 4 °C (Centrifuge 5804 R, Eppendorf AG ^®^, Hamburg, Germany).

### 4.4. Ventricular Hypertrophy

The hearts were removed; the right ventricle (RV) was detached and weighed, immediately frozen in liquid nitrogen and stored at −80 °C. RVH was determined by Fulton’s index (the ratio of the right ventricular weight (g) to the left ventricular plus septum weight (g)), as described previously [75]. Moreover, histological analyses were performed. Ventricular tissue was fixed in 4% paraformaldehyde at room temperature overnight and then dehydrated and embedded in paraffin. Paraffin-embedded tissue slices (5 µm thick) were routinely stained with hematoxylin-eosin (H&E) for evaluation of the morphology and sizes of cardiac cells under light microscopy. The cell areas (µm^2^) were measured with ImageJ software (ImageJ 1.48 v, National Institutes of Health, USA).

### 4.5. Lipid Peroxidation

Lipid oxidation in ventricular tissues was assessed through determination of malondialdehyde (MDA) concentrations (µmol/L) using a colorimetric assay. First, 30 mg of RV was homogenized in 400 µL of RIPA buffer (50 mM Tris-HCl, Triton X-100 1%, 150 mM NaCl, and 0.1% SDS) for 2 min at 4000 rpm with a homogenizer (Stir-Pak ^®^, Brinton, IL, USA) at 4 °C. Then, 100 µL of the sample (plasma or homogenized tissue) was mixed with 200 µL of trichloroacetic acid (TCA; 10%) on ice for 30 min. Subsequently, the mixture was centrifuged at 4000 rpm for 15 min at 4 °C, and the supernatant (200 µL) was mixed with 200 µL of thiobarbituric acid (TBA; 0.67%) and incubated in a water bath (100 °C) for 1 h. Finally, the absorbance was measured with a spectrophotometer (Thermo Electron Corporation ^®^, Madison, Wisconsin, USA) at 532 nm. To ensure the reliability of the results, a calibration curve with MDA at known concentrations was created prior to the analysis.

### 4.6. Hydrogen Peroxide (H_2_O_2_) Determination

Quantification of H_2_O_2_ in the RV was performed on day 30 through a colorimetric method with a Hydrogen Peroxide (H_2_O_2_) Assay Kit (Elabscience Biotechnology Co., Ltd. ^®^, Wuhan, China) and a microplate reader (Infinite ^®^200 PRO, TECAN ^®^, Mänedorf, Switzerland). The concentration of total protein was measured with a Bradford colorimetric method [76] with a BioPhotometer (Eppendorf AG ^®^, Hamburg, Germany) at 590 nm.

### 4.7. Western Blot Analysis

For protein extraction, 30 mg of tissue (RV) was first homogenized with 300 µL of RIPA lysis buffer containing a cocktail of phosphatase and protease inhibitors (4 mM PMSF, 10 μM leupeptin, 1 mM EDTA, 1 mM EGTA, 20 mM NaF, 20 mM HEPES, and 1 mM DTT). Then, the homogenates were centrifuged (Centrifuge 5804 R, Eppendorf AG ^®^, Hamburg, Germany) at 12,000 rpm for 20 min at 4 °C, and the supernatant was extracted. For quantification of total protein, the Bradford method was used [76] with a BioPhotometer (Eppendorf AG ^®^, Hamburg, Germany) at 590 nm. The protein extracts were stored at −80 °C. For Western blot analysis, the samples were diluted with 2× Laemmli buffer (0.125 M Tris-HCl, 4% (p/v) SDS, 20% (*v/v*) glycerol, 0.004% bromophenol blue, and 10% β-mercaptoethanol, pH 6.8). Proteins were separated according to their molecular weight (MW) under an electric field through sodium dodecyl sulfate polyacrylamide gel electrophoresis (SDS-PAGE) with 30% (*v/v*) bis-acrylamide. Then, the proteins were transferred from the SDS-PAGE gel to a polyvinylidene fluoride membrane (PVDF) with a semidry electroblotting system (OWL ^TM^ Separation Systems, Panther Semi-Dry Electroblotters, Thomas Scientific ^®^, Barrington, Ill, USA).

Once the PVDF membrane was blocked with bovine serum albumin (BSA), it was incubated with primary antibodies against LOX-1 (1:2000, no. ab60178, Lot. No. GR3250577-3, Abcam), p-Akt (1:2000, no. sc-33437, Lot. No. E0316, Santa Cruz Biotechnology ^®^), Akt (1:3000, no. sc-z8312, Lot. No. J2615, Santa Cruz Biotechnology ^®^), Nox2 (1:2000, no. sc-5827, Lot. No. B1214, Santa Cruz Biotechnology ^®^), Nox4 (1:4000, no. sc-55142, Lot. No. F1810, Santa Cruz Biotechnology ^®^), p38α MAPK (1:2000, no. sc-535, lot. No. C1113, Santa Cruz Biotechnology ^®^), p-p38α MAPK (1:2000, no. sc-7975-R, Lot. No. A1312, Santa Cruz Biotechnology ^®^), p22phox (1:4000, no. sc-20781, Lot. No. F1512, Santa Cruz Biotechnology ^®^), HIF-1α (1:2000, no. sc-10790, Lot. No. C2614, Santa Cruz Biotechnology ^®^), and β-actin (1:2000, no. sc-13065, Lot. No. K1418, Santa Cruz Biotechnology ^®^) overnight at 4 °C. p38α and Akt activation was determined as the ratio between the phosphorylated and nonphosphorylated forms and normalized to β-actin expression.

Finally, the membrane was incubated with the following secondary antibodies: anti-mouse (1:2000, no. sc-516102, Lot. No. A2219, Santa Cruz Biotechnology ^®^), anti-goat (1:2000, no. sc-2004, Lot. No. J0614, Santa Cruz Biotechnology ^®^) and anti-rabbit (1:2000, no. sc-2357, Lot. No. C2818, Santa Cruz Biotechnology ^®^) antibodies at dilutions in 3% BSA for 1 h at room temperature and visualized in a dark room with a chemiluminescence kit (Chemiluminescence West Pico ^®^, Super Signal Substrate, Thermo Scientific ^®^, Rockford, Ill, USA). Densitometry of the bands was performed with the ImageJ program and normalized to β-actin expression.

### 4.8. Data Analysis

All data were included in a database and analyzed using the SPSS program (IBM SPSS ^®^ V.21.0, Armonk, NY, USA). The normality of the variables was established with the Kolmogorov-Smirnov test; all variables were found to be normally distributed. The mean and standard error (SE) were calculated for each variable. To establish intergroup differences, Student’s t-test was used. The level of significance was *p* < 0.05.

## Abbreviations

RV	Right ventricle
CIH	Long-term chronic intermittent hypobaric hypoxia
RVH	Right ventricle hypertrophy
CIH	Long-term chronic intermittent hypobaric hypoxia
TAC	Transverse aortic constriction
NADPH oxidase	Nicotinamide adenine dinucleotide phosphate oxidase
LOX-1	Lectin-like oxidized low-density lipoprotein receptor-1
PKC	Protein kinase C
MEKK-2	MAP kinase kinase kinase-2
HIF-1α	Hypoxia-inducible factor-1α
H_2_O_2_	Hydrogen peroxide
MAPKs	Mitogen activated protein kinases
ROS	Reactive oxygen species
O_2_^.−^	Superoxide
OH	Hydroxyl radical
NX	Normobaric normoxia
BW	Body weight
Hct	Hematocrit
RV	Right ventricle
MDA	Malondialdehyde
TCA	Trichloroacetic acid
TBA	Thiobarbituric acid
BSA	Bovine serum albumin
